# 299. Systems immunology profiling of treatment-naïve children with multisystem inflammatory syndrome

**DOI:** 10.1093/ofid/ofac492.377

**Published:** 2022-12-15

**Authors:** Hong Zheng, Lael Yonker, Michele Donado, Ben Solomon, Brittany Boribong, Yael Gernez, Holden Maecker, Natalia Sigal, Bernard Kinane, Ann Arvin, Purvesh Khatri, Katja Weinacht

**Affiliations:** Stanford university, Stanford, California; Massachusetts General Hospital, Boston, Massachusetts; Stanford University, Stanford, California; Stanford University, Stanford, California; Massachusetts General Hospital, Boston, Massachusetts; Stanford University, Stanford, California; Stanford University, Stanford, California; Stanford University, Stanford, California; Massachusetts General Hospital, Boston, Massachusetts; Stanford University, Stanford, California; Stanford University, Stanford, California; Stanford University, Stanford, California

## Abstract

**Background:**

Multisystem inflammatory syndrome in children (MIS-C) is a severe post-infectious complication occurring weeks after SARS-CoV-2 infection. The exact mechanisms leading to immune dysregulation and organ damage remain incompletely understood. Progress in understanding the immunopathology underlying MIS-C has been halted by limited availability of pre-treatment patient samples and confounding effects of immunomodulatory treatment on previously studied specimens.

**Methods:**

In this study, we have restricted enrollment to treatment-naïve patients with MIS-C and used a systems biology approach combining CyTOF, single cell transcriptomics, serum cytokine profiling and T cell receptor sequencing to dissect how immune responses in children with MIS-C differ from children with mild SARS-CoV2 infection, adults with severe COVID-19 and healthy individuals. We also integrated single cell transcriptomics datasets from post-treatment MIS-C samples to study how immune responses change along disease course.

**Results:**

We identified increased activation markers and antigen presentation across multiple immune cell types in MIS-C patients from both CyTOF and single cell transcriptomics data. Importantly, in PBMCs of MIS-C patients, we identified a distinct subset of proinflammatory monocytes, with increased expression of interferon gamma response genes combined with a signature of enhanced complement expression, antigen processing and presentation, which was not observed in post-treatment MIS-C samples. Interestingly, this monocyte population bears resemblance to a subset of monocytes that emerges after the BNT162b2 mRNA vaccine booster. In addition, in PBMCs of MIS-C patients, we identify increased proportion of proliferating T/NK cells, suggesting distinct T cell expansions in MIS-C. T and NK cells in MIS-C samples also showed increased cell cytotoxicity markers.

**Conclusion:**

Taken together, treatment-naïve MIS-C samples display distinct monocyte clusters, activated antigen presentation and complement expression, and increased T and NK cell cytotoxicity, which may account for the clinical presentation of MIS-C.

**Disclosures:**

**Purvesh Khatri, PhD**, Cepheid, Inc.: Advisor/Consultant|Inflammatix, Inc.: Advisor/Consultant|Inflammatix, Inc.: Inflammatix is in negotiations to license the 9-gene signature discussed here.|Inflammatix, Inc.: Stocks/Bonds.

